# The 5′ untranslated region of the serotonin receptor 2C pre-mRNA generates miRNAs and is expressed in non-neuronal cells

**DOI:** 10.1007/s00221-013-3458-8

**Published:** 2013-03-15

**Authors:** Zhaiyi Zhang, Marina Falaleeva, Lily Agranat-Tamir, Amadis Pages, Eduardo Eyras, Joseph Sperling, Ruth Sperling, Stefan Stamm

**Affiliations:** 1 Department of Molecular and Cellular Biochemistry, University of Kentucky, 741 South Limestone, Lexington, KY 40536 USA; 2 Department of Genetics, The Hebrew University of Jerusalem, 91904 Jerusalem, Israel; 3 Universitat Pompeu Fabra, Dr. Aiguader 88, E08003 Barcelona, Spain; 4 Department of Organic Chemistry, The Weizmann Institute of Science, 76100 Rehovot, Israel

**Keywords:** miRNA, Alternative splicing, snoRNA, Serotonin receptor

## Abstract

The serotonin receptor 2C (HTR2C) gene encodes a G protein-coupled receptor that is exclusively expressed in neurons. Here, we report that the 5′ untranslated region of the receptor pre-mRNA as well as its hosted miRNAs is widely expressed in non-neuronal cell lines. Alternative splicing of HTR2C is regulated by MBII-52. MBII-52 and the neighboring MBII-85 cluster are absent in people with Prader–Willi syndrome, which likely causes the disease. We show that MBII-52 and MBII-85 increase expression of the HTR2C 5′ UTR and influence expression of the hosted miRNAs. The data indicate that the transcriptional unit expressing HTR2C is more complex than previously recognized and likely deregulated in Prader–Willi syndrome.

**Electronic supplementary material:**

The online version of this article (doi:10.1007/s00221-013-3458-8) contains supplementary material, which is available to authorized users.

## Introduction

The serotonin receptor 2C (HTR2C) is a G protein-coupled receptor located on the X chromosome. Binding studies indicate that the receptor is expressed exclusively in the brain, predominantly in neurons (Pompeiano et al. 1994). Mouse studies and the development of weight-loss drugs like Fen-Phen validated the serotonin receptor 2C (HTR2C) protein as an anti-obesity drug target (Miller 2005). In addition, HTR2C plays a central role in the regulation of mood and anxiety. It has also been implicated in depression, suicide, and schizophrenia (see Di Giovanni et al. 2011 for reviews). The HTR2C protein receptor is targeted by second generation anti-psychotics used for mania and depression, which block the receptor activity. One major side effect of these substances is weight gain (Lett et al. 2012), and there is genetic evidence that HTR2C is associated with the anti-psychotic-induced weight gain (Sicard et al. 2010). Direct investigation into the receptor function is limited, as it is expressed in neurons that are accessible only postmortem.

The receptor is encoded by a complex transcription unit spanning at least 326 kilobase pairs. Its pre-mRNA undergoes extensive processing that includes alternative splicing as well as editing of exon Vb, generating a total of 25 isoforms. Skipping of exon Vb generates a truncated protein isoform. Studies in transfected cells show that the truncated isoform forms a heterodimer with the full-length receptor, causing an entrapment in the endoplasmic reticulum and decrease of active cell surface receptor (Martin et al. 2012). The full-length protein isoforms are generated through editing and SNORD115 action. Central to the regulation of the HTR2C editing in exon Vb, is the formation of an extended stem-loop structure that is the substrate for RNA editing through ADAR2. The formation of the non-edited form is promoted by the non-coding RNA SNORD115 (MBII-52 in mouse). SNORD115 is a C/D box snoRNA that is further processed into smaller RNAs (Kishore et al. 2010; Falaleeva and Stamm 2013). SNORD115 binds to the double-stranded region that is regulated in exon Vb (Kishore and Stamm 2006b). The mouse and human orthologues of SNORD115 are almost identical (>90 % identity) and MBII-52 works on both human and mouse HTR2C reporter constructs (Kishore and Stamm 2006a). SNORD115 is absent in patients with Prader–Willi syndrome, and mouse models indicate that a deregulation of the serotonergic system contributes to the disease (Morabito et al. 2010; Doe et al. 2009).

**Fig. 1:**
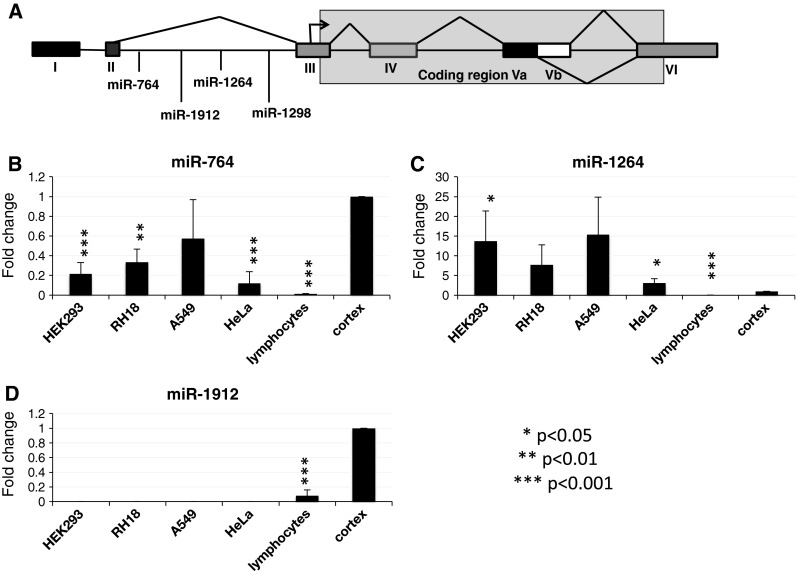
Expression of miRNAs located in serotonin receptor intron 2 in different cell lines. **a** Schematic diagram of the serotonin receptor 2C pre-mRNA. **b**–**d** qRT-PCR showing amplification of miRNAs. The amount of miRNAs in human cortex tissue was set to one, and miRNA abundance in other cells was expressed as a fraction of the expression in cortex

In contrast to the well-studied coding region of the HTR2C receptor pre-mRNA, little is known of the extended 5′ untranslated region of the receptor pre-mRNA that contains two introns and three exons (Fig. 1a), which host miRNAs. Mouse models employed to study HTR2C function still contain the 5′ UTR and the hosted miRNAs (Tecott et al. 1995; Xu et al. 2008), and it is therefore possible that the mouse knockouts capture only the protein function of the HTR2C transcriptional unit, but not its function as a host of miRNAs.

miRNAs are 22nt long non-coding RNAs. The majority of miRNAs reside in pre-mRNA, both in the UTRs and in introns. In the nuclear pre-mRNA, miRNAs are part of extended double-strand structures forming pri-miRNAs that are recognized and cleaved by DROSHA/DGCR8, forming pre-miRNA that are exported into the cytosol. In the cytosol, DICER forms mature miRNAs that are loaded on argonaute proteins. miRNAs act in gene regulation by binding to mRNA, where they can block translation, cause RNA decay, RNA cleavage, as well as chromatin silencing (Esteller 2011).

Here, we report that the HTR2C gene expresses exons 1–3 and miRNAs located in intron 2 in non-neuronal cell lines, demonstrating that part of the HTR2C gene is expressed outside the brain and in non-neuronal cell types. The abundance of the encoded miRNAs is regulated by MBII-52 and MBII-85, two RNAs implicated in Prader–Willi syndrome. Since miRNAs encoded by HTR2C intron 2 are broadly expressed and detectable in lymphocytes, they contribute to the gene function in non-neuronal cells and could allow to monitor the response of the HTR2C gene to drug treatments.

## Materials and methods

### RNase protection

HEK 293T cells were transiently transfected with plasmids coding for MBII-52 and MBII-85 snoRNA (mouse orthologous of SNORD115 and SNORD116 respectively). Total RNA was isolated from cells using Trizol LS reagent (Invitrogen) according to manufactory protocol.

As a probe, we used a uniformly labeled RNA against mouse chr7:59,520,283–59,520,365 for MBII-52 and chr7:59,861,729–59,861,827 for MBII-85, as previously described (Shen et al. 2011).

RNAs from human cortex was isolated using the RNeasy Lipid Tissue Kit (Qiagen, Hilden) according to manufactory protocol.

### Cell culture

The HEK293, HeLa, and A549 cells were obtained from the American Type Culture Collection (ATCC). RH18 cells were obtained from St. Jude Children’s Research Hospital, and the primary human fibroblast cells were obtained from Coriell Institute (GM 00498D). All the cells were grown in the recommended medium containing 10 % fetal bovine serum (FBS) at 37 °C under 5 % CO_2_. Lymphocytes were collected from the blood of a healthy male donor and were purified using ACCUSPIN System-Histopaque-1077 (Nishimura et al. 2007).

### RT-PCR

Sixty nanograms of total RNA (30 ng/μl), 5 pmol of reverse primer, and 40 U of SuperScript III reverse transcriptase (Invitrogen) were mixed in 5 μl of RT (reverse transcription) buffer. To reverse transcribe the RNA, the reaction was incubated at 55 °C for 50 min. Half of the RT reaction was used for cDNA amplification. The reaction was performed in 25 μl and contained 10 pmol of specific forward and reverse primers, 200 μM dNTPs, 1× Taq polymerase buffer, and 1 U of Taq DNA polymerase. The amplification was performed in an Eppendorf PCR System with the following conditions: initial denaturation for 4 min at 94 °C, optimized number of cycles (30 cycles for cDNAs from cortex and for β-actin; 40 cycles for 5HT2C cDNAs from cells) for 30 s at 94 °C, 30 s at 55 °C and an extension of 45 s at 72 °C. After the last cycle, the reaction was held for 5 min at the extension temperature to complete the amplification of all products.

### Brain tissues

Cortex tissues were obtained postmortem from three patients with Prader–Willi syndrome and three individuals as a control group. The tissue was obtained from the NICHD brain bank in Maryland. A detailed analysis of the tissues was performed in (Falaleeva et al. 2013).

### TaqMan analysis

Thirty nanograms of total RNA, 1× RT primer, 15 mM dNTPs, 50 U of SuperScript III Reverse Transcriptase, 1× RT (Reverse Transcription) buffer, 4 U of RNase inhibitor were mixed in a 15 μl RT reaction. The reverse transcription was performed under the following condition: 30 min at 16 °C, 30 min at 42 °C, and 5 min at 85 °C.

One-fifth of the RT reaction was used for the qPCR. The reaction was performed in 20 μl and contained 1× TaqMan MicroRNA assay primer, 1× TaqMan universal PCR Master Mix no AmpErase UNG (Applied Biosystems). The amplification was carried out in a Stratagene Mx3000p Thermocycler with the following conditions: initial denaturation for 10 min at 95 °C, 40 cycles for 15 s at 95 °C, and an extension of 1 min at 60 °C.

### RNA isolation and deep sequencing

RNA was extracted from supraspliceosomes prepared from frozen HeLa cells (CILBIOTECH), as previously described (Azubel et al. 2006). The integrity of the RNA was evaluated by an Agilent 2100 bioanalyzer. For small RNA library construction, ~10 mg of RNA was used, following the Illumina Directional mRNA-Seq Library Prep. (Pre-release Protocol) with the following changes: (1) The poly-A selection and fragmentation of mRNA steps were omitted; (2) To enrich for small RNAs, ethanol precipitation was used instead of column fractionation of the PNK-treated RNA. Adaptors were then ligated to the 5′ and 3′ ends of the RNA, and cDNA was prepared from the ligated RNA and amplified to prepare the sequencing library. The amplified sequences were purified by PAGE and sequences representing RNA smaller than 200 nt were extracted from the gel. The library was sequenced using the Genome AnalyzerIIX System by Illumina. The sequencing data, after filtering the adaptors and low-quality sequences, were aligned to mirBase.

For RNAse protection assays and miRNA analysis, RNA was isolated by Trizol LS reagent, and total RNA (including small RNAs and ribosomal RNAs) was used.

### Search of MBII-85 and MBII-52 targets in the serotonin receptor 2C (HTR2C) pre-mRNA

The HTR2C pre-mRNA sequence was downloaded from ENSEMBL release 54. Subsequently, for each psnoRNA construct from MBII-52 and MBII-85, all the possible substrings with base pairing with any substring of the HTR2C genomic sequence were searched exhaustively. The following criteria were used as follows: (a) The substrings were at least 15 nucleotides long; (b) Up to 2 mismatches were allowed; (c) Non-canonical G-U base-pairings were also considered.

## Results

### Detection of miRNAs located in the 5′ UTR by deep sequencing

During investigation of the splicing regulation of the serotonin receptor 2C, we performed deep sequencing of non-coding RNA from supraspliceosomes (Sperling et al. 2008) isolated from HeLa cells. The entire repertoire of nuclear pre-mRNAs are individually packaged in splicing active supraspliceosomes, that likely represent the in vivo composition of spliceosomes (Sperling et al. 2008). The sequencing data showed the expression of miR-764, miR-1260, miR-1912, and miR-1298. These four miRNAs are located in the 5′ UTR of the serotonin receptor 2C pre-mRNA, between exon 2 and 3 (Fig. 1a). miR-1298 was most abundant with 4217 reads, followed by miR-1264 with 2560 reads, miR-764 with 817 reads, and miR-1912 with 522 reads. miR-764, miR-1264, miR-1912, and miR-1298 are only found in the serotonin receptor 2C pre-mRNA. Since the expression of the serotonin receptor 2C is considered neuron-specific, these results were highly surprising.

### Expression of miRNAs in cell lines

We next validated the deep-sequencing findings using qRT-PCR employing available TaqMan probes for miR-764, miR-1912, and miR-1264 (there is no probe available for miR-1298). We tested total RNA from several different cell lines: HEK293 cells (fibroblasts), RH18 cells (rhabdomyosarcoma), A549 (lung cancer, adenocarcinoma), HeLa (cervical cancer), primary lymphocytes (from male blood), and human cortex. As shown in Fig. 1b, d, we could detect miR-764 and miR-1264 in all cell lines tested, but miR-1912 only in primary lymphocytes. As expected, all miRNAs are expressed in human brain (cortex), considered to be the only expressing tissue (Canton et al. 1996). Unexpectedly, miR-1264 is expressed at higher ratios in cell lines than in cortex. The qPCR analysis is in agreement with the read count of the deep-sequencing data and indicated an expression pattern of miR-1264 > miR-764 > miR1912.

### The 5′ untranslated region of serotonin receptor 2C gene is widely expressed

**Fig. 2:**
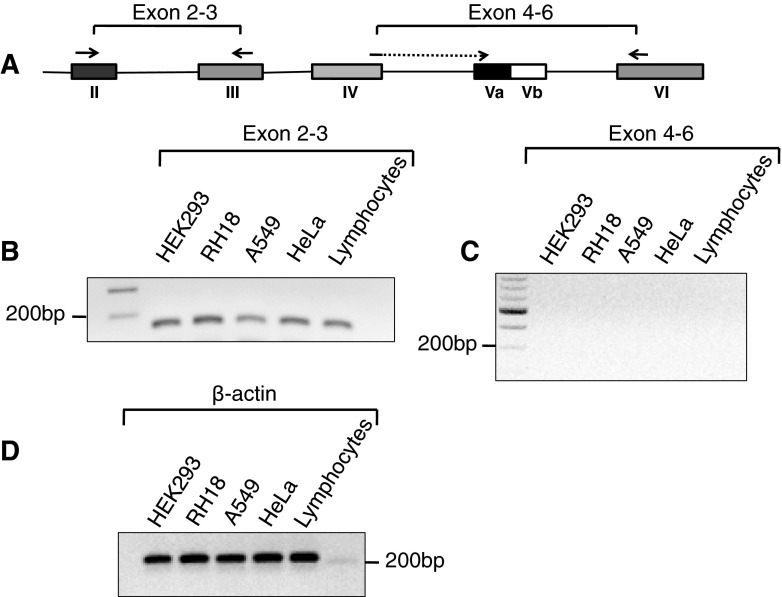
Expression of hosting exons for miRNAs. **a** Schematic illustration of the primers used for amplification. **b** RT-PCR amplification of intron 2 hosting the miRNAs. Primers in exon 2 and 3 indicated in **a** were used. **c** RT-PCR amplification of the coding region of the HT2CR using primers in exon 4 and 6. **d** RT-PCR amplification of β-Actin, demonstrating equal loading

We next determined the expression of the 5′ untranslated pre-mRNA region of serotonin receptor 2C (Fig. 2a) that hosts the miRNAs in intron 2. Using flanking primers in exon two and three, we observed amplification in all cell lines tested. In contrast, there was no amplification when we used primers in exons four and six (Fig. 2b, c). These data indicate that the HT2CR generates two mRNAs, one corresponding to the 5′ UTR (exons 1–4) and one corresponding to the full-length RNA (exons 1–6). The long mRNA composed of exons 1–6 has previously been detected in Northern Blots (Canton et al. 1996) using brain tissue. The existence of shorter mRNAs corresponding to the 5′ UTR is supported by an expressed sequence tag, DC317996, which encompasses exon 1–4. In the absence of an open reading frame, the function of the mRNA corresponding to exons 1–4 is likely to host several miRNAs.

### Changes of miRNAs in brains of subjects with Prader–Willi syndrome (PWS)

The serotonin receptor 2C undergoes alternative pre-mRNA splicing in exon V. The inclusion of exon Vb is promoted by processed snoRNAs generated from the MBII-52 cluster (Kishore and Stamm 2006b; Falaleeva and Stamm 2013). Data from mouse systems indicate that this regulation occurs in vivo (Doe et al. 2009). We therefore asked whether MBII-52 influences the expression of miRNAs located in HT2CR intron 2.

**Fig. 3:**
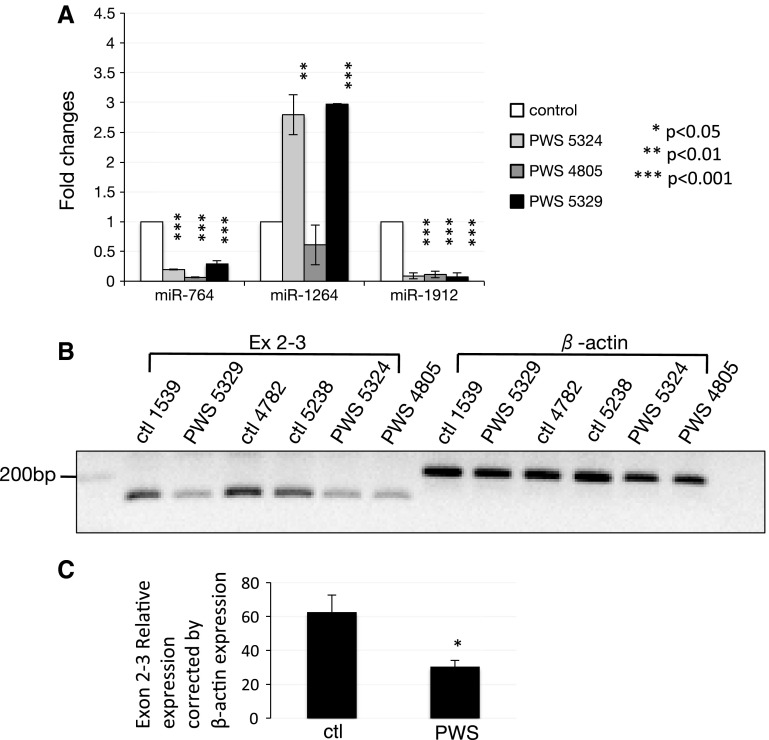
Changes of miRNAs in PWS samples. **a** qRT-PCR results using cortex samples from controls and PWS patients. **b** RT-PCR of intron 2, β-actin was used as a loading control. **c** Quantification of data from panel **b**. The means and SD are shown

First, we analyzed tissue samples obtained from patients with PWS and compared them to controls. Patients with PWS do not express SNORD115/MBII-52 and SNORD116/MBII-85. In all cases, similar frontal cortical regions were used. As shown in Fig. 3a, PWS patients express less miR-764 and miR-1912 than controls but express more miR-1264 than controls.

Next, we determined the expression of the exons flanking the hosting intron and found them significantly lower expressed than the exons from controls (Fig. 3b).

The data suggest that miRNA expression could be influenced by the lack of SNORD115/116 RNAs in Prader–Willi syndrome subjects.

### Transfection of MBII-52 and MBII-85 changes abundance of miRNAs expressed from intron 2

**Fig. 4:**
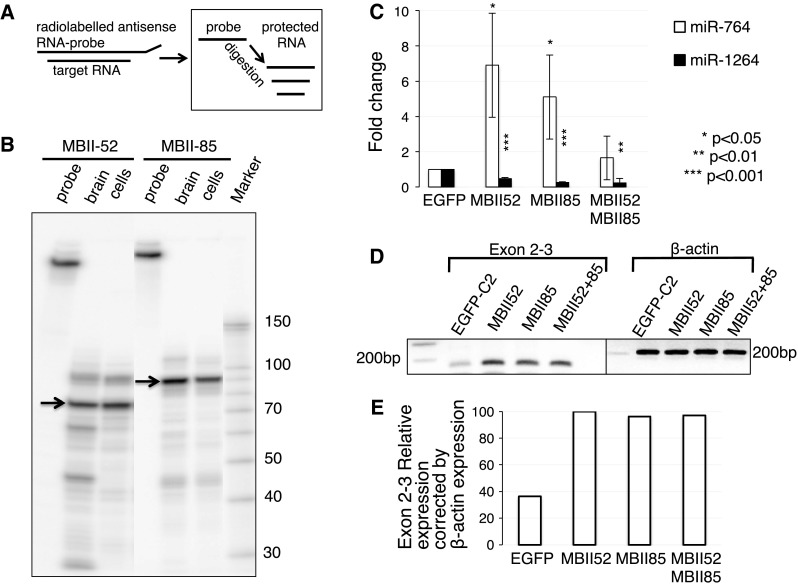
Influence of MBII-52 and MBII-85 overexpression on miRNAs. **a** Schematic illustration of the RNase protection assay. The RNA:RNA hybrid formed between in vitro transcribed radiolabeled RNA and target RNA is shown (*left*). Treatment with RNase A/T1 removes single-stranded regions and leaves protected psnoRNAs (*right*). **b** Expression of MBII-52 and MBII-85 snoRNAs in HEK293T cells and mouse brain. HEK293 cells were transfected with snoRNAs expression constructs (1 μg plasmid per 1 ml media). Total RNA was isolated from mouse brain (brain) and HEK293 cells (cells) after 42 h of transfection and analyzed by RNase protection assay as described (Shen et al. 2011). *Arrows* show full-length snoRNAs, additional *lower bands* show snoRNAs processing products. **c** RT-qPCR amplification of miRNAs encoded in intron 2. Total RNA was extracted from HEK293 cells transfected with control plasmid-expressing EGFP-C2, MBII-52 or MBII-85 snoRNAs (*n* = 4). **d** RT-PCR amplification of the flanking intron 2, and the amplification of β-actin mRNA as loading control. **e** Quantification of RT-PCR results shown on 4D. Exon 2 and 3 expression normalized to the expression of β-actin and plotted on a *chart*

To test the influence of MBII-52 and MBII-85 on miRNA abundance directly, we transfected HEK293 cells with expression constructs generating MBII-52 and MBII-85. The expression of MBII-52 and MBII-85 was measured by RNase protection analysis (Fig. 4a, b). We then measured the abundance of miR-764, miR-1264, and miR-1912 by qRT-PCR. As shown in Fig. 4c, miR-764 was strongly upregulated by expression of MBII-52 and MBII-85. In contrast, miR-1264 was downregulated. miR-1912 could not be detected. These changes mirror the situation in PWS subjects, where miR-764 is down and miR-1264 is upregulated.

Next, we determined the influence of MBII-52 and MBII-85 on the expression of the hosting intron using RT-PCR that amplifies the flanking exons 2–3. As shown in Fig. 4d (quantified in Fig. 4e), we found an upregulation of the exons caused by the overexpression of both psnoRNAs.

Together, the data suggest an influence of MBII-52 and MBII-85 on the expression of miR-764 and miR-1264 and the hosting part of the gene.

### Target sequences of MBII-52 and MBII-85 in the serotonin receptor pre-mRNA

**Fig. 5:**
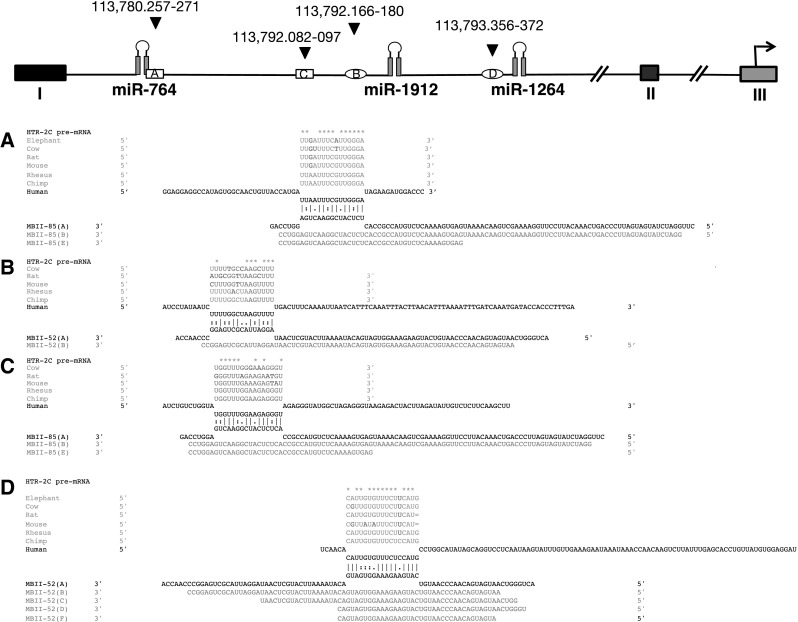
Bioinformatically predicted binding sites of MBII-52 and MBII-52 located next to miRNAs in intron 2. The *top line* shows a schematic representation of intron 2. *Numbers* indicate the coordinates in human genome built (Hg19). psnoRNA-binding sites are indicated by *small boxes*; *square*: MBII-85, *oval*: MBII-52. **a**–**d** Alignment and phylogenetic conservation of the binding sites. The human sequences closest to the mouse sequences are indicated with their copy numbers in Hg19 after SNORD115 and SNORD116 (shown in *red*) (color figure online)

We analyzed binding sites for MBII-52 and MBII-85 on the serotonin receptor pre-mRNA. Very little is known so far about how psnoRNAs bind to their targets. Accordingly, we performed an unbiased, exhaustive search of potential targets of MBII-52 and MBII-85 psnoRNA constructs on the serotonin receptor 2C (HTR2C) gene. We initially searched for at least 15 nt matches and allowed up to two mismatches between psnoRNA sequences and the serotonin receptor 2C pre-mRNA. As shown in Fig. 5, we detect binding sites for MBII-52 and MBII-85 on the receptor 2C pre-mRNA, flanking the pre-miRNA stem-loop structure. All putative binding sites are visualized on the UCSC browser, under http://regulatorygenomics.upf.edu/Projects/htr2c-targets.html.

As shown in Fig. 5a–d, most of these binding sites are in evolutionary conserved regions. Importantly, the binding sites are conserved between the mouse and human copies. However, there are numerous binding sites in the intron as well. Therefore, the exact mechanism remains to be determined.

## Discussion

### HTR2C gives rise to mRNA widespread expression

Unexpectedly, we found that intron 2 of the serotonin receptor 2C is expressed in all cell lines investigated, which is in contrast to the protein-coding region of the HTR2C gene. The most likely explanation is the presence of a transcriptional termination signal prior to exon 4 that is not used in neurons. In contrast to other cells, neurons will therefore express the full-length mRNA encoding the protein. This notion is supported by ESTs encompassing only the first three exons and by the observation that HTR2C mRNA from brain has the predicted size of one long mRNA containing exons one to six. Furthermore, databases indicate the presence of CpG islands only in exon I, suggesting that there is only one transcriptional start site. The HTR2C transcriptional unit is therefore more complex than previously thought.

The expression of HTR2C intron 2 could have practical use, as it offers the possibility to monitor HTR2C expression under various pharmacological treatments that target the encoded protein. Numerous gene systems show a regulation between the 3′ UTR or encoded proteins and gene expression. Given the pharmacological importance of HTR2C, it will be interesting to investigate whether the miRNAs change during treatment with drugs that target HTR2C protein.

### miRNAs encoded by HTR2C have different regulation

The 5′ UTR of the HTR2C gene functions to host the miRNAs. Whereas intron 2 expression is comparable between cell lines, the miRNAs show differences in expression, which suggest a further regulation, either a cell-type-specific processing of the pre-miRNA or a cell-type-specific stability.

The function of these miRNAs and their regulation remains to be determined. Mir-764-5p promotes osteoblast differentiation as it translationally represses Hsc70-interacting protein/STIP1 homology and U-Box containing protein 1 (CHIP/STUB1), which promotes osteoblast differentiation (Guo et al. 2012). Although there are no experimentally validated targets for the other miRNAs, it is almost certain that they control other cellular processes, which adds to the function of the serotonin receptor 2C pre-mRNA. These functions were not captured by knock-out mice that removed only the coding region, but left the genomic region encoding intron 2 intact (Tecott et al. 1995; Xu et al. 2008). The knock-out mice were mostly studied with regard to changes in food intake, and it will be interesting to analyze whether the intron 2-encoded miRNAs can modify the phenotype or are deregulated in these knock-out mice.

### psnoRNAs from the MBII-52 and MBII-85 loci influence the expression of encoded miRNAs

Inclusion of the serotonin receptor’s alternative exon Vb is promoted by processed snoRNAs from the MBII-52 cluster (Kishore and Stamm 2006b; Kishore et al. 2010). We therefore tested whether the two psnoRNAs, MBII-52 and MBII-85, influence expression of miRNAs encoded in intron 2. Transfection studies showed that both psnoRNAs increase abundance of the exons flanking intron 2, suggesting an influence on the pre-mRNA. This finding is supported by the analysis of postmortem material, where we found less exon change to two/three expression in samples from PWS patients who do not express these psnoRNAs.

The effect of psnoRNAs on the encoded miRNAs is more complicated, as miR764 is promoted by MBII-52 and MBII-85, whereas miR1264 is repressed. This interdependency is reflected in samples from PWS subjects, but the mechanism for the different regulations is not clear. We also noticed that one patient (4805) expressed less mir-1264 than controls, which is different from the other PWS subjects tested. The reason for this difference is not clear.

Nonetheless, our data show that a deregulation of micro-RNAs located in intron 2 of the serotonin receptor 2C likely contribute to the Prader–Willi syndrome phenotype.

## Electronic supplementary material

Below is the link to the electronic supplementary material.

Supplementary material 1 Table 1: List of primers used (XLSX 47 kb)
